# A randomised phase II study of second-line XELIRI regimen versus irinotecan monotherapy in advanced biliary tract cancer patients progressed on gemcitabine and cisplatin

**DOI:** 10.1038/s41416-018-0138-2

**Published:** 2018-06-29

**Authors:** Yi Zheng, Xiaoxuan Tu, Peng Zhao, Weiqin Jiang, Lulu Liu, Zhou Tong, Hangyu Zhang, Cong Yan, Weijia Fang, Weilin Wang

**Affiliations:** 1 Cancer Biotherapy Centre, the First Affiliated Hospital of Zhejiang University, Hangzhou, 310003 China; 2 Department of Hepatobiliary Pancreatic Surgery, the First Affiliated Hospital of Zhejiang University, Hangzhou, 310003 China

**Keywords:** Biliary tract cancer, Chemotherapy

## Abstract

**Background:**

The majority of advanced biliary tract cancer (ABTC) patients will progress after gemcitabine and cisplatin (GP) doublet therapy, while the standard second-line regimen has not been established. We conducted this study to assess the efficacy and safety of second-line irinotecan and capecitabine (XELIRI) regimen vs. irinotecan monotherapy in ABTC patients progressed on GP.

**Methods:**

Sixty-four GP refractory ABTC patients were randomised to either irinotecan 180 mg/m^2^ on day 1 plus capecitabine 1000 mg/m^2^ twice daily on days 1–10 of a 14-day cycle (XELIRI-arm) or single-agent irinotecan 180 mg/m^2^ on day 1 of a 14-day cycle (IRI-arm). Treatments were repeated until disease progression or unacceptable toxicity occurred.

**Results:**

A total of 60 patients were included in the analysis. For XELIRI and IRI-arms, respectively, the median PFS was 3.7 vs. 2.4 months, 9-month survival rate 60.9% vs. 32.0%, median OS 10.1 vs. 7.3 months, and disease control rate 63.3% vs. 50.0%. The most common grade 3 or 4 toxicities were leucopaenia and neutropaenia.

**Conclusions:**

This randomised, phase II study of irinotecan-containing regimens in good PS second-line ABTC patients showed a clear benefit of XELIRI regimen over irinotecan monotherapy in prolonging PFS, with acceptable toxicity.

## Introduction

Biliary tract cancer (BTC) is a rarely^1^ and highly fatal malignancy with a 5-year overall survival (OS) rate of only about 10% for cholangiocarcinoma and less than 5% for gallbladder cancer.^2,3^ Radical resection is the only potentially curative approach to early stage BTC. However, recurrence after surgical resection is common since BTC has high potential and propensity to metastasise. Moreover, at the time of diagnosis, most of the patients present with advanced stage disease which precludes the possibility of surgical resection. Therefore, palliative chemotherapy is the standard therapeutic option for advanced biliary tract cancer (ABTC).

In the past decades, several studies have demonstrated the efficacy and safety of gemcitabine in ABTC.^4,5^ In 2010, the combination regimen with gemcitabine plus cisplatin (GP) was shown to significantly improve the survival of patients with ABTC compared to gemcitabine alone as first-line therapy in two randomised trials (OS: 11.7 vs. 8.1 months, 11.2 vs. 7.7 months, respectively).^6,7^ GP doublet therapy is now the standard first-line regimen for ABTC.

Unfortunately, the majority of ABTC cases will eventually progress on GP doublet. Approximate 50% of these patients with good performance status (PS) undergo further treatment,^8,9^ but no consensus has been made for the most suitable regimen in the second-line setting.^10–12^ There were several studies evaluating the potential effects of irinotecan as a monotherapy or as part of a combination in ABTC, and its anti-tumour effect was encouraging with acceptable toxicity.^13–16^ More recently, capecitabine has shown a survival benefit over observation alone for adjuvant therapy in BTC in a phase III randomised trial, with little reported impact on quality of life.^17^ However, the role of irinotecan and capecitabine (XELIRI) regimen in second-line ABTC chemotherapy remains an unresolved issue.

Given the promising results from the previous studies, we conducted this study to compare the efficacy and safety of second-line XELIRI regimen to irinotecan monotherapy in good PS ABTC patients with progressive disease following GP doublet chemotherapy, as a randomised prospective phase II study. The primary objective of this study was to compare the progression-free survival (PFS) in ABTC patients who received one of these two therapies. The secondary objectives were OS, response rate (RR) and assessment of safety.

## Materials and Methods

### Study design

This was a single-centre, randomised phase II study to evaluate the efficacy and safety of XELIRI regimen compared with irinotecan monotherapy in GP doublet refractory, good PS ABTC patients. Patients were randomised to either single-agent irinotecan 180 mg/m^2^ on day 1 of a 14-day cycle (IRI-arm) or irinotecan 180 mg/m^2^ on day 1 plus capecitabine 1000 mg/m^2^ twice daily on days 1–10 of a 14-day cycle (XELIRI-arm). The assigned treatment was delivered until disease progression, unacceptable toxicity or patient refusal.

### Eligibility criteria

Patients were eligible for the study if they were 18 years of age or older and had a histologically confirmed diagnosis of locally advanced or metastatic biliary tract adenocarcinoma (intrahepatic or extrahepatic cholangiocarcinoma, and gallbladder carcinoma) and a radiologically confirmed progression after first-line GP doublet chemotherapy, an Eastern Cooperative Oncology Group (ECOG) PS of 0 or 1. Other eligibility criteria were radiological measurable disease, adequate function of major organs, in particular a haemoglobin ≥10 g per 100 ml, white blood cells ≥3000/mm^3^, neutrophils ≥1500/mm^3^, platelets ≥80,000/mm^3^, total bilirubin levels ≤2 times the upper limit of the normal range, liver-enzyme (alanine aminotransferase (ALT)/aspartate aminotransferase (AST)) levels ≤5 times the upper limit of the normal range, renal function with levels of serum creatinine ≤1.5 times the upper limit of the normal range, and a calculated glomerular filtration rate ≥45 ml/min.

This study (ClinicalTrials.Gov ID: NCT02558959) was approved by Ethics Committee of the First Affiliated Hospital of Zhejiang University and was conducted according to the Declaration of Helsinki and guidelines on Good Clinical Practice. Written informed consent was obtained from each patient before random assignment.

### Efficacy and safety assessment

All patients who received at least one dose of the study drug were included in the efficacy and safety assessment. Medical records of each patient were reviewed to collect relevant data on demographics, tumour characteristics, surgery, biliary stenting, and serum levels of carbohydrate antigen 19-9 (CA 19-9) one-day before the start of second-line treatment. Tumour response was assessed in each patient every 6 weeks by means of computed tomography or magnetic resonance imaging according to the Response Evaluation Criteria in Solid Tumour (RECIST, version 1.1). PFS was defined as the time interval between the initiation of the second-line chemotherapy and disease progression or death, whichever occurred first. Toxicities were graded according to the National Cancer Institute’s CTCAE v4.0. Information on third-line chemotherapy after disease progression was also collected.

### Statistical analysis

The sample size was calculated by the selection method of Simon et al.,^18^ which is based on the previous reports and the assumption that XELIRI regimen could prolong the PFS for 1.2 months than irinotecan monotherapy.^15,16^ With these assumptions, 30 patients per arm were needed to appropriately select the combination therapy with a probability of ≥80%. PFS and OS were estimated using the Kaplan–Meier method. Surviving patients without disease progression were censored at the end of follow-up (January 2018). A Cox proportional hazards model was used to calculate the hazard ratio (HR), 95% confidence interval (CI) and its two-tailed *P*-value. Fisher’s exact test was used to compare the patient characteristics, response and disease control rates, and toxicities between the two treatment arms. A two-sided *P-*value less than 0.05 was considered significant. All statistical analyses were performed with SPSS software (version 21.0; IBM Corporation, Armonk, NY, USA).

## Results

### Patients

**Fig. 1:**
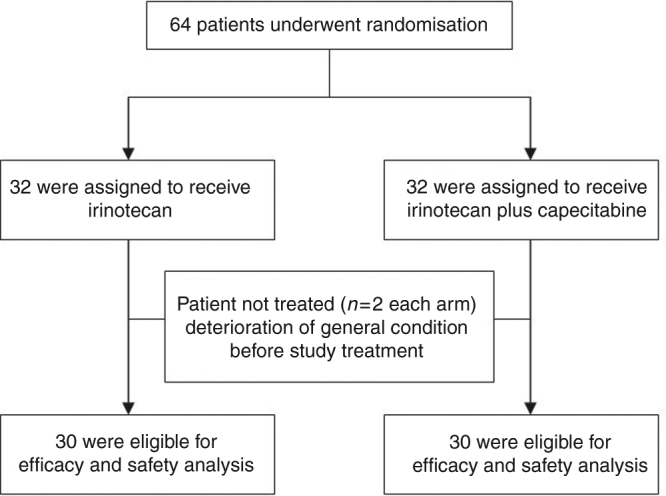
Patient enrolment, randomisation and treatment

This study was carried out in the First Affiliated Hospital of Zhejiang University from September 2015 to September 2017. Sixty-four patients were randomised to either XELIRI-arm or IRI-arm. Each arm has two patients not treated because of the early deterioration of general condition before study treatment. All of the remaining 60 patients, 30 in the XELIRI-arm and 30 in the IRI-arm, received at least one dose of study treatment. Efficacy and safety were evaluated for each of these 60 patients (Fig. 1). Baseline characteristics (Table 1) were well balanced between the two arms.

**Table 1:** Baseline characteristics of the patients

Characteristic	XELIRI (*n*=30)	IRI (*n*=30)	*P*-value
	*n* (%)	*n* (%)	
*Gender*			
Male	16 (53.3)	19 (63.3)	
Female	14 (46.7)	11 (36.7)	0.432
*Age (year)*			
Median	54	55	0.979^a^
Range	26-70	40-68	
*ECOG PS*			
0	24 (80.0)	25 (83.3)	
1	6 (20.0)	5 (16.7)	0.739
*Extent of disease*			
Locally advanced	14 (46.7)	16 (53.3)	
Metastatic	16 (53.3)	14 (46.7)	0.606
*Primary tumour site*			
Intrahepatic bile duct	20 (66.7)	21 (70.0)	
Extrahepatic bile duct	3 (10.0)	4 (13.3)	
Gallbladder	7 (23.3)	5 (16.7)	0.779
*Biliary stenting*			
Yes	3 (10.0)	2 (6.7)	
No	27 (90.0)	28 (93.3)	0.64
*Previous surgery*			
Yes	24 (80.0)	26 (86.7)	
No	6 (20.0)	4 (13.3)	0.488
*First-line PFS (months)*			
Median	6.7	6.9	0.527^a^

*XELIRI* irinotecan and capecitabine, *IRI* irinotecan,

*ECOG PS* Eastern Cooperative Oncology Group performance status.

^a^*t*-Test

### Treatment compliance

A total of 159 and 137 cycles were administered at the time of analysis in the XELIRI-arm and IRI-arm, respectively. At the end of the first 8 weeks, treatment compliance was similar in the two groups, with 56.7% receiving four cycles of XELIRI and 60.0% receiving four cycles of irinotecan alone; however, in the treatment period overall, more patients in the IRI-arm discontinued treatment prematurely, primarily because of disease progression. In the first 8 weeks of treatment, an average of 91% of the planned dose was delivered to patients in the XELIRI-arm, as compared with 89% in the IRI-arm; however, in the second 8 weeks, the average was 85% in the XELIRI-arm as compared with 67% in the IRI-arm.

### Efficacy

**Fig. 2:**
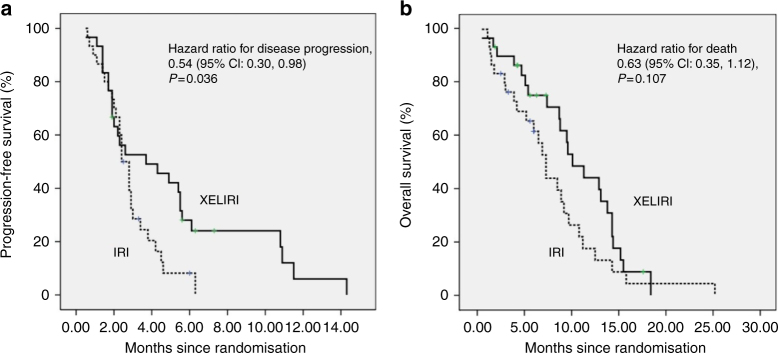
Kaplan–Meier curve of progression-free survival and overall survival. **a** Progression-free survival. **b** Overall survival. XELIRI irinotecan and capecitabine combination, IRI irinotecan monotherapy, CI confidence interval

A total of 60 patients were evaluable for tumour response according to the protocol, 30 in the XELIRI-arm and 30 in the IRI-arm. In total, two complete responses (CR) were observed in the XELIRI-arm, but no CR was observed in the IRI-arm (6.7% vs. 0%). Besides, both arms had 2 patients achieved partial response (PR) (6.7% vs. 6.7%). The RR was higher in XELIRI-arm (13.7% vs. 6.7%, *P* = 0.389). In addition, 15 patients had stable disease (SD) in the XELIRI-arm, while 13 patients in the IRI-arm had SD (50.0% vs. 43.3%). The disease control rate (CR + PR + SD) was 63.3% vs. 50.0% in favour of the combination therapy (*P* = 0.297). The median PFS (3.7 months vs. 2.4 months, *P* = 0.036) and 9-month survival rate (60.9% vs. 32.0%, *P* = 0.045) were better for the XELIRI-arm compared to IRI-arm. However, the prolonged OS (10.1 months vs. 7.3 months, *P* = 0.107) was not statistically significant (Fig. 2, Table 2).

**Table 2:** Summary of efficacy: overall response and survival

Parameter	XELIRI (*n*=30)	IRI (*n*=30)	*P*-value
	*n* (%)	*n* (%)	
*Overall response rate*			
Complete response (CR)	2 (6.7)	0 (0.0)	
Partial response (PR)	2 (6.7)	2 (6.7)	
Stable disease (SD)	15 (50.0)	13 (43.3)	
Progressive disease (PD)	11 (36.7)	15 (50.0)	
Response rate (RR)	13.3%	6.7%	0.389
Disease control rate (DCR)	63.3%	50.0%	0.297
*Progression-free survival (PFS)*			
Median PFS (95% CI)	3.7 months (0.3, 7.1)	2.4 months (2.0, 2.8)	0.036^*^
Hazard ratio (95% CI)	0.54 (95% CI: 0.30, 0.98)		
*Overall survival (OS)*			
9 months survival rate	60.9%	32.0%	0.045^*^
Hazard ratio (95% CI)	0.30 (95% CI: 0.09, 0.99)		
Median OS (95% CI)	10.1 months (7.4, 12.8)	7.3 months (6.1, 8.5)	0.107
Hazard ratio (95% CI)	0.63 (95% CI: 0.35, 1.12)		

*XELIRI* irinotecan and capecitabine, *IRI* irinotecan, *CI* confidence interval.

^*^P < 0.05

For prognostic factor analysis, the survival of ABTC patients with CA 19-9 ≥400 IU/ml was worse than that of patients with CA 19-9 <400 IU/ml. Moreover, the PFS were longer with XELIRI combination in non-gallbladder cancer patients (4.9 vs. 2.4 months), as well as in patients with CA 19-9 <400 IU/ml (4.3 vs. 2.8 months), as shown in Table 3.

**Table 3:** Prognostic factor analysis of progression-free survival time

Parameter	Median progression-free survival time (months) (95% CI)		*P*-value
	XELIRI (*n*=30)	IRI (*n*=30)	
*Tumour site*			
Gallbladder	2.3 (1.3, 3.3)	2.8 (1.9, 3.7)	0.689
Non-gallbladder	4.9 (0.7, 9.1)	2.4 (1.8, 3.0)	0.032^*^
*CA 19-9*			
≥400 IU/ml	2.3 (0.1, 5.0)	2.4 (0.5, 4.3)	0.341
<400 IU/ml	4.3 (0.1, 9.6)	2.8 (2.4, 3.2)	0.022^*^
*Age*			
≥60	2.6 (0.1, 7.0)	2.4 (1.8, 3.0)	0.079
<60	2.0 (0.1, 5.7)	2.4 (1.7, 3.1)	0.124
*Extent of disease*			
Locally advanced	2.2 (0.9, 3.5)	2.4 (1.7, 3.1)	0.203
Metastatic	3.7 (0.1, 7.6)	2.4 (2.2, 2.6)	0.053

*XELIRI* irinotecan and capecitabine, *IRI* irinotecan, *CI* confidence interval.

^*^P < 0.05

### Safety

All adverse events recorded in this study were predictable and manageable based on the safety profile of irinotecan and capecitabine. Table 4 describes the main adverse events which occurred during the study. Generally, toxicities were mild: the most common events (≥50%) were leucopaenia, neutropaenia, nausea, anaemia and hand–foot syndrome in the XELIRI-arm, and leucopaenia, neutropaenia, nausea, thrombocytopaenia and anaemia in the IRI-arm. The incidence of hand–foot syndrome and stomatitis was higher in the XELIRI-arm, which is common in capecitabine-containing regimens. No treatment related death occurred. Most of the patients recovered from the adverse events by supportive care or reducing the dose intensity.

**Table 4:** Summary of adverse events^a^ associated with chemotherapy

Adverse events	XELIRI (*n*=30)		IRI (*n*=30)	
	Grade 3/4, *n* (%)	All grades, *n* (%)	Grade 3/4, *n* (%)	All grades, *n* (%)
*Haematological*				
Leucopaenia	8 (26.7%)	28 (93.3%)	8 (26.7%)	27 (90.0%)
Neutropaenia	8 (26.7%)	25 (83.3%)	7 (23.3%)	26 (86.7%)
Thrombocytopaenia	2 (6.7%)	13 (43.3%)	1 (3.3%)	15 (50.0%)
Anaemia	2 (6.7%)	20 (66.7%)	3 (10.0%)	21 (70.0%)
*Gastrointestinal*				
Diarrhoea	1 (3.3%)	8 (26.7%)	1 (3.3%)	7 (23.3%)
Nausea	3 (10.0%)	27 (90.0%)	2 (6.7%)	25 (83.3%)
Vomiting	1 (3.3%)	10 (33.3%)	1 (3.3%)	12 (40.0%)
*General and laboratory*				
Hand–foot syndrome	2 (6.7%)	16 (53.3%)	0 (0%)	0 (0%)
Stomatitis	0 (0%)	5 (16.7%)	0 (0%)	3 (10.0%)
Liver enzyme elevation	0 (0%)	10 (33.3%)	0 (0%)	8 (26.7%)

*XELIRI* irinotecan and capecitabine, *IRI* irinotecan.

^a^Events were graded according to CTCAE v4.0

### Post-study chemotherapy

Among the patients who went on to receive third-line therapy, 13 patients (43.3%) in the XELIRI-arm received post-study chemotherapy including docetaxel (8 patients) and S-1 (5 patients). In the IRI-arm, 11 patients (36.7%) received post-study chemotherapy including capecitabine (4 patients), docetaxel (6 patients), and S-1 (1 patient).

## Discussion

The treatment beyond disease progression after GP doublet chemotherapy in ABTC patients remains a challenge. Until now, three independent systematic reviews have provided the most comprehensive results regarding the use of second-line chemotherapy in ABTC.^19–21^ However, the survival data reported in these studies were not satisfying.

Although there was no solid evidence that indicates any clear survival benefit of the use of second-line chemotherapy, irinotecan had been preliminary evaluated as a monotherapy or as part of combination therapies in ABTC, and the median OS for second-line treatment was approximately 6–8 months.^15,16,22^ Since all the patients recruited in this study preserved a good PS and require continuing care, it was reasonable and ethical to choose irinotecan monotherapy as control. However, whether there is a definite survival advantage of irinotecan-containing regimens over irinotecan monotherapy is still unclear. Capecitabine is another active agent which had been substantially studied for the anti-tumour effect in ABTC patients.^11,23–25^ More recently, capecitabine had established its fundamental role as the standard adjuvant therapy in BTC for a median OS of 53 months without significant impairment of quality of life (BILCAP study).^17^ Thus, XELIRI regimen was chosen as the study scheme.

On the basis of considerations above, we started this randomised, phase II study to evaluate the anti-tumour activity and safety of XELIRI regimen over irinotecan monotherapy in GP refractory ABTC patients. In the present study, although the increase in OS was not significant, XELIRI regimen prolonged the second-line PFS of ABTC patients by 1.3 months, increased the 9-month survival rate by approximate 30% over irinotecan alone (all *P* < 0.05). The relatively high RR and two cases with CR in the XELIRI-arm were also impressive. More importantly, the survival benefit in this study was achieved with the addition of an outpatient capecitabine schedule without an increase of hospitalisation. These data provided evidence that XELIRI regimen is an effective second-line treatment option for ABTC patients when compared to irinotecan monotherapy.

Although the BICC-C study found CapeIRI regimen (irinotecan 250 mg/m^2^ on day 1, capecitabine 1000 mg/m^2^ twice daily on days 1–14, every 3 weeks) was more toxic that impeded its acceptance as an active regimen in metastatic colorectal cancer,^26^ the entire toxicity profile observed in this study was similar between the two treatment arms, only the incidence of hand–foot syndrome and stomatitis was increased in the XELIRI-arm, which was inevitable for the addition of capecitabine. We believe the dose adjustment of irinotecan and capecitabine from a 3-week schedule to a 2-week schedule may contribute to the better tolerance of this combination. Actually, several studies using a dose-adjusted irinotecan and capecitabine regimen had also revealed a better tolerance and efficacy in metastatic colorectal cancer.^27–29^ Therefore, optimising the dose intensity and administration scheme would be of great importance in maximising the efficacy of irinotecan and capecitabine combination, regardless of tumour origin.

There were several studies focusing on the selection of ABTC patients who might benefit from second-line chemotherapy. Parameters including better ECOG PS, lower serum CA 19-9 level, longer PFS after first-line chemotherapy, and less metastatic organs are considered better prognostic factors for ABTC in this setting.^30,31^ In this study, we also studied the prognostic factors including primary site, CA 19-9 level, age, and extent of disease, as shown in Table 3; patients with higher serum CA 19-9 level showed worse PFS than those with lower serum CA 19-9 level, this being consistent with previous reports.^31^ It is also important to note that the XELIRI regimen showed longer PFS in patients with serum CA 19-9 level <400 IU/ml and non-gallbladder origin, suggesting these two subgroup patients are more likely to benefit from the XELIRI combination therapy.

There were also several limitations to this study. First, as BTC is still a rather rare cancer, and the study was planned and conducted at a single centre, the sample size in this study was relatively small, which could have selection bias. Second, more extrahepatic cholangiocarcinoma patients with jaundice and extensive disease patients with poor PS were excluded according to the eligibility criteria; thus, the proportions of intrahepatic cholangiocarcinoma and locally advanced disease patients were relatively higher in this study. Third, according to recent studies, single agent capecitabine may served as a better second-line control arm, but at the time of our study design, the results of BILCAP study was far before reported. Therefore, according to the institute’s experience and literature review, irinotecan was chosen as control arm. A phase III study of larger data sets with multi-centre participation is warranted in the future.

In conclusion, this is the first randomised, phase II study to evaluate the efficacy and safety of irinotecan-containing regimens in good PS second-line ABTC patients. This study met its primary objective, and the outcomes from this study showed a clear advantage of XELIRI regimen over irinotecan monotherapy in prolonging PFS, with acceptable toxicity. XELIRI regimen merits further evaluation in large-population, multi-centre phase III trials in ABTC.

## Availability of data and materials

All data are fully available without restriction.
